# Does environmental management system certification affect green innovation performance?—Based on a moderated mediating effects model

**DOI:** 10.3389/fpsyg.2023.1264207

**Published:** 2024-01-08

**Authors:** Jinsong Zhang, Mengmeng Wang, Muyao Li

**Affiliations:** Accounting School, Harbin University of Commerce, Harbin, China

**Keywords:** environmental management system certification, social responsibility disclosure, green innovation performance, traditional innovation performance, moderated mediating effect

## Abstract

What is the impact of environmental management system certification on green innovation performance, and is it a futile endeavor or a profitable one? Grounded in the principles of ecological civilization construction and green development, this study embarks on a comprehensive examination. Initially, it investigates the varying impacts of environmental management system certification on both traditional innovation performance and green innovation performance. Subsequently, it dissects the underlying mechanisms and moderating factors influencing the latter, including an exploration of intermediary effects. The empirical findings of this study are as follows: (i) Environmental management system certification emerges as a catalyst for innovation performance, with the primary impact observed in the realm of green innovation performance. (ii) Social responsibility disclosure is identified as a mediating factor in the relationship between environmental management system certification and green innovation performance. (iii) Larger enterprises, those equipped with robust equity incentives, and those operating in less competitive markets are more prone to benefit from the impact of environmental management system certification on social responsibility disclosure. This, in turn, amplifies the promotion of green innovation performance. However, the moderating effect of property rights on the mediating path remains statistically insignificant. (iv) Environmental management system certification exerts a more pronounced influence on green innovation performance in regions characterized by lower economic development. Moreover, it particularly stimulates exploratory green innovation performance, surpassing its impact on exploitative green innovation performance.

## 1 Introduction

Currently, China is undergoing a transformative phase, aiming to transcend its previous low-end position within the global value chain and attain high-quality economic development. The adoption of an innovation-driven development strategy has emerged as the path forward. The conventional long-term economic growth model has inflicted considerable harm on the ecological environment. Environmental issues have not only impacted the quality of life but have also posed as impediments to economic and social progress (Wang et al., 2020). In the Fifth Plenary Session of the 18th CPC Central Committee, a set of five major development concepts—“innovation, coordination, green, openness, and sharing”—were introduced for the first time, elevating green development to an unprecedented level of importance. Nevertheless, while the significance of green development is apparent, translating these principles into action proves challenging. This challenge is exemplified by China's ranking of 120th in the annual Global Environmental Performance Index by Yale University in 2018, underscoring the severity of environmental pollution and the inadequacy of environmental regulation in China. Confronted with the dual imperatives of green development and fostering innovation, the Party Central Committee, during the Sixth Plenary Session of the 19th Central Committee, reiterated the critical strategic importance of ecological civilization construction. Green technology innovation is widely recognized as a pivotal means of reconciling the dilemmas of environmental protection, pollution prevention, resource recovery, and economic growth (Xiaoxiao and Juntao, 2021; Li and Lu, 2023). Moreover, it empowers enterprises to embrace the mission of environmental stewardship while reaping the benefits of innovation outcomes. While command-based environmental regulations can mitigate the adverse environmental effects of enterprise activities within the innovation sphere, it is challenging to surmount the ecological civilization construction bottleneck solely through external coercive policies. Thus, China has taken a proactive stance by introducing environmental management system certification, bridging gaps, and granting enterprises greater flexibility and autonomy. Subsequently, command-based and voluntary environmental regulations have synergized. Porter's hypothesis corroborates that environmental regulations can generate “innovation compensation effects” through mechanisms like “product compensation” and “production process compensation.” These effects effectively offset the compliance costs associated with environmental regulations, affirming the innovation-driven role of command-based environmental regulations. Nonetheless, the relationship between environmental management system certification, a form of voluntary environmental regulation, and innovation performance remains a pertinent question. Additionally, given that green innovation represents a distinct form of technological innovation within the context of ecological civilization, the extent to which green innovation performance reflects the impact of environmental management system certification is an open query. The formation of the transmission mechanism and the factors influencing it remain areas requiring further exploration, as current studies do not provide sufficient depth on these topics. Addressing these questions is crucial for realizing the five development concepts.

This study contributes significantly in three key dimensions: Firstly, it augments the body of literature on environmental management system certification in China. It does so by conducting an in-depth exploration of the impact of environmental management system certification on innovation performance. Simultaneously, it analyzes the differential effects on traditional innovation performance and green innovation performance within the framework of sustainable development, thereby deepening our understanding of green innovation performance. Secondly, this study delves into the mediating role of social responsibility disclosure based on stakeholder theory and signaling theory. This fills a void in existing research that has not thoroughly examined indirect influence mechanisms. The study mitigates endogeneity concerns by employing instrumental variable methods and further validates the existence of the mediating path using bootstrap models. Furthermore, it applies a moderated mediation model to explore how firm characteristics, nature, internal governance, and external environmental factors moderate these paths. This aids firms in tailoring their environmental management strategies effectively in various contexts. Lastly, the study conducts a heterogeneity analysis to assess the effects of environmental management system certification on both exploratory and exploitative green innovation performance. Additionally, it examines the role of environmental management system certification in green innovation performance across regions with varying degrees of economic development. This analysis sheds light on the inner workings and conditions of environmental management system certification effectiveness. In summation, this study significantly advances our comprehension of the intricate interplay between environmental management system certification and innovation performance, offering valuable insights for both scholars and practitioners.

## 2 Literature review

Given the increasing significance of environmental management system (EMS) certification among companies, scholars have initiated investigations into the antecedents and outcomes of this certification. Numerous internal and external factors influencing corporate certification have been explored, encompassing environmental and ethical motivations, management perceptions, governmental regulations, competitive dynamics, stakeholder pressures, and more. As the number of certifications has grown, studies have shifted their focus to analyze the consequences of EMS certification, primarily with respect to environmental performance (Graafland, 2018; Erauskin-Tolosa et al., 2020), economic performance (Arocena et al., 2020; Wang and Mao, 2020), and its impact on innovation activities. Research findings concerning the first two types of performance have been mixed, revealing both positive and negative effects (Heras-Saizarbitoria et al., 2020; Zhang et al., 2021). For instance, Erauskin-Tolosa et al. (2020) demonstrated that ISO 14001 and EMAS certification had a positive influence on corporate environmental performance. Arocena et al. (2020) found that ISO 14001 certification led to a reduction in carbon intensity and improved profitability. A substantial portion of the academic literature has centered on the ISO 14001 market and environmental benefits. Treacy et al., adopting a practice-based perspective and an event study approach derived from prior ISO 14001 research, determined that corporate adoption of ISO 14001 resulted in significant enhancements in employee productivity, fixed asset turnover, return on assets, and operating performance (He and Shen, 2019). Nonetheless, it is worth noting that environmental management system certification entails not only benefits but also costs. Scholars such as Miroshnychenko et al. (2017) and Riaz and Saeed (2020) identified negative impacts of ISO 4001 on corporate performance. Unlike environmental and financial performance, green technological innovation stands out as the most comprehensive and fundamental solution for addressing environmental pollution (Su et al., 2022). Consequently, studies exploring the relationship between environmental management system certification and corporate innovation have primarily concentrated on green innovation, yielding more consistent conclusions.

This study posits that, in line with the principles of ecological civilization construction and green development, the resolution to environmental management issues must be sought through green innovation. There exist gaps in the analysis of the impact pathways of environmental management system certification on enterprises' green innovation performance within existing literature. The effects of indirect impact pathways in distinct contexts remain largely unexplored in prior research. This study endeavors to provide empirical evidence and illuminate the mechanisms that underlie the effectiveness of environmental management system certification.

## 3 Mechanistic analysis and hypotheses

### 3.1 Environmental management system certification and corporate innovation performance

According to neoclassical economics, environmental regulation, while effective in controlling the environmental impact of enterprises, necessitates resource allocation for environmental management. This allocation increases the cost of pollution control and results in a “crowding-out effect” on R&D funds, which is detrimental to green technological innovation and hinders the enhancement of a firm's competitiveness (Kemp and Pontoglio, 2011). In contrast, scholars like Porter argue that environmental regulation and economic development can be mutually reinforcing. While environmental regulation may increase short-term operational costs for businesses, it also incentivizes innovation activities, leading to additional returns that offset the costs of environmental management. This theory, known as the “Porter hypothesis,” has injected vitality into the field of environmental management and innovation. Scholars have scrutinized the validity of the Porter hypothesis across various contexts, challenging the notion that environmental regulation is confined to mandatory government directives and passive compliance by enterprises. Over time, the concept of environmental regulation has evolved to encompass a blend of mandatory and voluntary practices. Even voluntary environmental regulation has demonstrated its capacity to empower enterprises with greater initiative (Ni et al., 2019).

Environmental management system certification, as a voluntary environmental regulatory tool, guides companies in implementing and enhancing their environmental management systems (Ni et al., 2019). Many countries actively endorse voluntary environmental policies. For instance, the U.S. government stipulates that companies establishing effective environmental management systems, such as ISO 14001 certification, may receive reduced penalties from the Environmental Protection Agency when environmental regulations are violated (Daddi et al., 2015). Certification signifies a company's capability to comply with pertinent environmental standards and requirements (Mosgaard and Kristensen, 2020). Drawing on the concept of the innovation compensation effect, a certified company's environmental management processes effectively control key environmental factors, optimize resource allocation, and establish a foundation for innovation activities (He and Shen, 2019). Hicks' theory of induced innovation suggests that environmental management, by increasing input costs, compels companies to adopt technological innovation as a solution (Cai et al., 2020). Environmental management system certification acts as a catalyst for enterprise innovation activities, facilitating improved innovation performance. Based on the above analysis, the following hypotheses are proposed:

H1a: Environmental management system certification positively influences enterprise innovation performance.

However, traditional innovation activities often disregard environmental concerns in their processes, making them susceptible to resource and environmental crises. In contrast, green technology innovation seamlessly integrates economic and ecological benefits within the constraints of resource and environmental sustainability. As the principles of sustainable development are increasingly implemented in practice, traditional technological innovation is gradually giving way to green technological innovation. Green innovation activities inherently prioritize environmental conservation and are well-supported by resources (Wiengarten et al., 2017). This alignment with the ethos of environmental management system certification leads businesses to favor green innovation as a means of addressing the challenges posed by environmental management (Zhang et al., 2023). Green innovation performance can effectively offset the short-term negative impact of environmental management costs on profitability. Consequently, the contribution of environmental management system certification to traditional innovation performance is comparatively weaker than its impact on green innovation performance. In summary, environmental management system certification has a positive influence on enterprise innovation performance, with its primary impact being on green innovation performance. Subsequent research will accordingly focus on green innovation performance. In summary, the following hypothesis is proposed:

H1b: Environmental management system certification has a more pronounced positive effect on enterprise green innovation performance compared to traditional innovation performance.

### 3.2 Environmental management system certification, social responsibility disclosure and corporate green innovation performance

Stakeholder theory, rooted in the concept of pressure, asserts that growing concern for environmental issues among stakeholder groups, including government entities, consumers, suppliers, and employees, has exerted mounting public pressure on companies. Consequently, organizations are increasingly compelled to balance the maximization of shareholder value with the fulfillment of environmental obligations. Failure to meet these environmental responsibilities exposes companies to public criticism regarding environmental pollution, unsafe products, and more. When a company achieves environmental management system certification, it signifies compliance with international environmental standards. These standards encompass the establishment and implementation of an environmental management system in accordance with regulations, successful completion of initial assessments, and thorough planning for the execution of the environmental management system. Drawing on the insights of signaling theory and reputation theory, certified companies, in a bid to shield themselves from adverse market reactions, engage in the disclosure of information regarding their social responsibility commitments. This disclosure underscores their dedication to fulfilling social responsibilities, portraying them as responsible and dedicated corporate entities in the eyes of consumers (Imed et al., 2020; Lu et al., 2020; Paweł et al., 2021). Simultaneously, it communicates to the public a strong alignment between corporate philosophy and social values (Valenciano-Salazar et al., 2021). This alignment endears them to the external influencers of corporate green innovation—stakeholder decisions—thus reducing financial risks associated with green innovation activities and enhancing their green innovation performance (Lu et al., 2020; Bai et al., 2021). The role of environmental management system certification in promoting social responsibility disclosure, and consequently bolstering green innovation performance, establishes the potential for enterprises to achieve a dual victory encompassing both environmental preservation and economic prosperity. For example, Haier Group, a global leader in the electrical appliance industry, has exemplified its commitment to social responsibility through environmental management system certification. By doing so, it has demonstrated its integration of environmental protection principles and energy-efficient technologies into product design. This strategic approach has enabled the company to realize green technological innovation, particularly in the development of fluorine-free products. As a result, Haier Group has gained a significant foothold in the blue ocean market of electrical appliances, reaping not only economic benefits but also substantial environmental advantages. In cases like that of Haier Group, environmental management system certification does not impede enterprise development; rather, it serves as a catalyst for the disclosure of corporate social responsibility, enhancing green innovation performance and unlocking new profit opportunities in the process. In light of the above analysis, the following hypothesis is proposed:

H2: Environmental management system certification enhances corporate green innovation performance by promoting social responsibility disclosure.

### 3.3 Analysis of moderated mediating effects based on firm size

In the context of corporate Environmental Management System (EMS) certification influencing green innovation performance through social responsibility disclosure, the size of the firm assumes a pivotal role in determining the availability of resources for executing environmental management and subsequent certification. The cost associated with undertaking EMS certification can be substantial (Frondel et al., 2018). Smaller enterprises embarking on EMS certification might grapple with resource constraints, or worse, could perceive EMS certification as a mere symbolic gesture in response to customer and competitor pressures regarding environmental strategies. This symbolic approach often translates into a lack of confidence in disclosing social responsibility information. In contrast, larger corporations are better positioned to embrace EMS certification in earnest, integrating the environmental management framework seamlessly into their day-to-day operations and demonstrating proactive engagement in social responsibility disclosure. There are several reasons behind this trend. First, the implementation of an operational framework for EMS certification involves significant costs and time investments. When viewed through the lens of traditional cost theory, which operates in accordance with the principle of economies of scale, it becomes evident that production costs for enterprises tend to decrease as their scale expands until reaching an optimal point (O'Reilly et al., 2023). Large-scale enterprises possess the requisite resource levels to manage these costs efficiently and maintain lower marginal expenses, thereby facilitating the efficient implementation of environmental management system certification. The study conducted by González et al. (2008) also indicated that larger enterprises exhibit greater efficiency in implementing practices aimed at reducing material costs. Secondly, larger companies attract more attention, and their actions, or lack thereof, are subject to heightened scrutiny and monitoring by the public. Any irresponsible conduct on their part is more likely to be exposed and condemned by the media and other stakeholders. Consequently, such actions entail greater political costs for the company and can result in the deterioration of the company's image and its relationships with stakeholders. This, in turn, can stifle green innovation activities. Therefore, larger enterprises have a greater capacity to eliminate the occurrence of superficial and insincere certification processes. Their heightened enthusiasm for social responsibility disclosure consequently leads to improved green innovation performance. In essence, the size of the enterprise exerts a moderating influence on the process of environmental management system certification, driving social responsibility disclosure, and subsequently enhancing green innovation performance. Building upon the above analysis, the following hypothesis is posited:

H3: The larger the firm size, the more it can enhance the impact of environmental management system certification on social responsibility disclosure, thus amplifying its influence on green innovation performance.

### 3.4 Analysis of moderated mediating effects based on the nature of ownership

The distinct characteristics of property rights have given rise to discernible variations in corporate management practices (Caselli and Figueira, 2023). To examine whether the impacts of different property rights attributes, in the context of advancing social responsibility disclosure through environmental management system (EMS) certification and consequently augmenting green innovation performance, are consistent, this study conducts an analysis of the moderating effects of property rights attributes. This analysis is conducted through the lens of property rights theory and is rooted in the concept of property rights heterogeneity. Firstly, it's important to note that non-state-owned enterprises (non-SOEs) exhibit a slight advantage over state-owned enterprises (SOEs) in terms of resource utilization efficiency and the decision-making process. In the Chinese context, SOEs often grapple with inefficiencies in decision-making and possess redundant assets. Due to their direct government backing, SOEs are more inclined to utilize their advantageous resources for green innovation activities. The assurance of government support diminishes the necessity for SOEs to acquire additional innovation resources through the disclosure of social responsibility information. In contrast, non-SOEs contend with relatively limited resources and lack robust government support. Consequently, non-SOEs place greater emphasis on the benefits stemming from environmental management. Secondly, the phenomenon of soft budget constraints prevalent among SOEs often leads to a lack of innovative dynamism. Property rights theory posits that private enterprise owners enjoy the right to residual profits, instilling in them a strong incentive to continuously enhance organizational efficiency. Thus, in terms of profit incentives, private enterprises surpass traditional SOEs. Non-SOEs are more motivated to seek opportunities for realizing innovative value, and they possess a greater incentive to implement environmental protection strategies. This motivation prompts them to disclose social responsibility information through EMS certification, creating a reservoir of valuable resources to propel green innovation performance within the organization. Therefore, the nature of property rights assumes a moderating role in the process of propelling social responsibility disclosure through EMS certification, thereby intensifying its impact on green innovation performance. Building upon the above analysis, the following hypothesis is proposed:

H4: In comparison to state-owned enterprises, non-state-owned enterprises are more inclined to witness the promotion of social responsibility disclosure through environmental management system certification, resulting in a more pronounced effect on green innovation performance.

### 3.5 Analysis of moderated mediating effects based on equity incentives

Management, occupying a pivotal position as corporate decision-makers, wields significant authority in the realm of corporate governance and exercises decisive control over voluntary environmental regulation, specifically environmental management system certification (EMS). By drawing on the principles of principal-agent theory and the economic man hypothesis, it becomes evident that management's stance toward green innovation is largely contingent on the delicate balance between private costs and private benefits. Green innovation initiatives inherently carry a degree of risk. While shareholders advocate for corporate engagement in innovative endeavors to maximize overall corporate value, managers frequently shy away from these investments due to their short-term self-interest horizons. Within the framework of principal-agent theory, rooted in contract theory, it is posited that endowing executives with a certain level of equity within the firm can effectively tether their income to the firm's surplus, serving as a potent motivator for increased effort and commitment (Ma and Wang, 2022). Consequently, the interests of shareholders and the utility of executives become intricately entwined, giving rise to a mechanism that both shares benefits and risks (Chen et al., 2023). Equity incentives, to a significant extent, intimately connect managerial wealth with the future valuation of the company (Fabrizi, 2014). In response, companies deploy concerted management efforts to enhance the firm's share price and bolster its reputation through the implementation of equity incentives (Assaf and Saleh, 2021). Moreover, a more pronounced degree of equity incentives corresponds to an intensified capacity of EMS certification to stimulate social responsibility disclosure. This, in turn, fosters an enhancement in green innovation performance and serves to alleviate agency problems associated with innovation, which often arise due to conflicting interests between shareholders and management (Albert et al., 2021). Based on the comprehensive analysis provided, the following hypothesis is postulated:

H5: The stronger the equity incentive the more it promotes the enhancing effect of environmental management system certification on social responsibility disclosure, and thus on green innovation performance.

### 3.6 Analysis of moderated mediating effects based on the intensity of market competition

Market competition, serving as an external environmental factor, engenders a developmental paradigm characterized by “survival of the fittest” and heightens the moral hazard dilemma faced by enterprises. To ascertain whether disparities exist in the impact of environmental management system certification, mediated by social responsibility disclosure, on green innovation performance across varying levels of market competition intensity, this study delves into the moderating influence of market competition intensity on this intermediary pathway. The liquidation threat hypothesis posits that intensified competition elevates the risk of bankruptcy and liquidation, exerting substantial pressure on firms to refocus their developmental objectives toward enhancing firm performance (Adamolekun et al., 2022). Within markets characterized by heightened competition, the heightened comparability among firms and the concomitant reduction in monitoring costs compel management to prioritize the capture of market share and the fortification of their competitive standing. This, in turn, often results in an unabated pursuit of profits, relegating environmental management to the periphery and fostering an environment where socially responsible behavior is sidelined. Consequently, the likelihood of fostering the disclosure of social responsibility information through environmental management system certification diminishes. Conversely, in markets characterized by less cutthroat competition, enterprises are less inclined to sacrifice the ecological environment solely for survival. Instead, the market environment becomes conducive to the cultivation of a corporate reputation rooted in environmental responsibility. The pursuit of environmental management system certification and the subsequent disclosure of social responsibility are perceived as advantageous endeavors that enhance corporate standing and confer benefits upon enterprises. Consequently, these enterprises are more inclined to engage in environmental management and social responsibility disclosure, ultimately resulting in an augmentation of their green innovation performance. It is evident that within less competitive markets, environmental management system certification and social responsibility disclosure assume a more central role in corporate priorities, stimulating greater corporate commitment to green innovation. Drawing from the above analysis, the following hypotheses are posited:

H6: Diminished market competition intensity positively correlates with an augmented influence of environmental management system certification on social responsibility disclosure, consequently amplifying its impact on green innovation performance.

**Figure 1:**
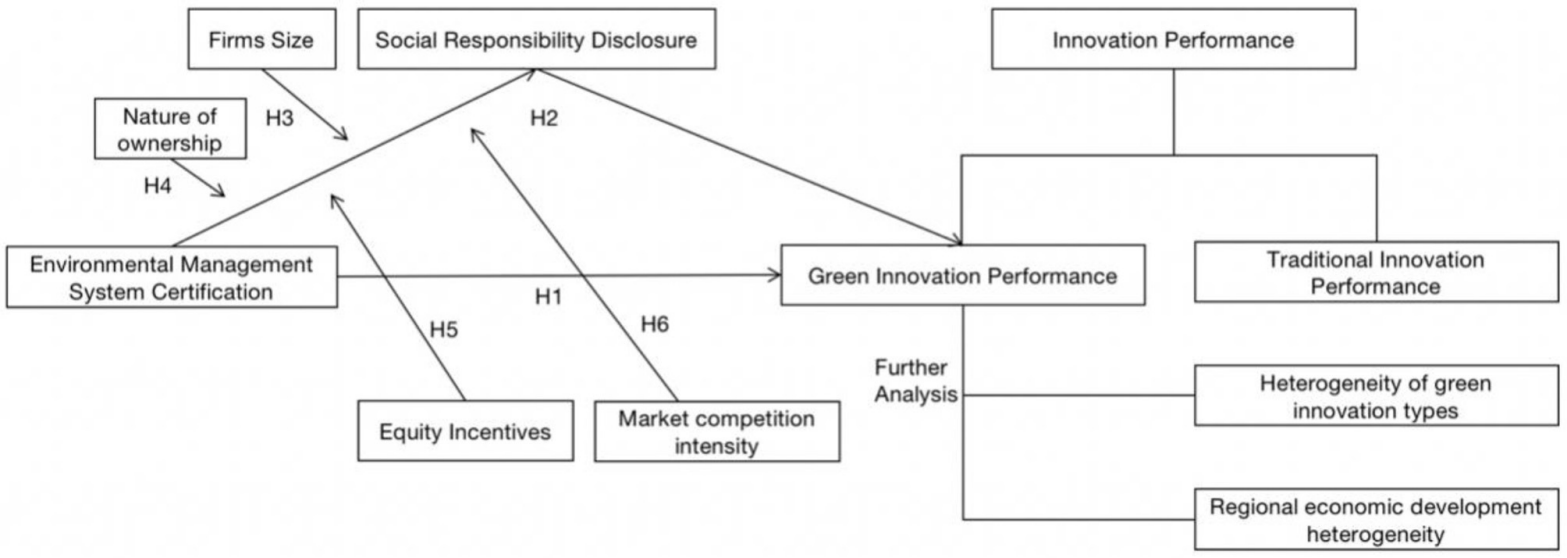
Conceptual model of the main research content.

In summary, the conceptual model of this paper is shown in Figure 1.

## 4 Data and empirical model

### 4.1 Data collection and the sample

The concept of Ecological Civilization Construction and Green Development was first introduced during the Fifth Plenary Session of the 18th CPC Central Committee in 2012. Subsequently, it gained national prominence, and environmental protection and green development became top priorities. Furthermore, the global outbreak of COVID-19 occurred after 2020. To minimize potential interference with this research, the study focused on the period between 2012 and 2020.The choice of the Shanghai and Shenzhen A-share markets as the research context is based on several considerations. Firstly, these markets are among the largest and most influential stock markets in China, making them highly representative of the nation's economic landscape. Secondly, the vast number of companies listed on the Shanghai and Shenzhen A-share markets spans a wide range of industries and sectors, ensuring a comprehensive and extensive dataset for more effective research. Moreover, these stock markets are significantly influenced by Chinese government policies and regulations, which hold substantial sway over the nation's economic dynamics. Exploring these markets offers valuable insights into the operation and development of the Chinese economy. Consequently, this study focused on Shanghai and Shenzhen A-share listed companies that disclosed patent applications between 2012 and 2020 as the initial sample. The sample was further refined by excluding financial and insurance companies and eliminating enterprises categorized as ST and ^*^ST. To mitigate the impact of extreme values, all continuous variables were Winsorized at the 1% upper and lower levels. Data on social responsibility fulfillment was sourced from the social responsibility scores of listed companies on Hexun.com, while green patent data was obtained from the CNRDS database. All other data used in the study was extracted from the Guotai'an (CSMAR) database.

### 4.2 Definition of main variables

#### 4.2.1 Explained variables

Innovation Performance (Tpatant), Traditional Innovation Performance (Bpatant), and Green Innovation Performance (Gpatant): This study differentiates between traditional and green innovation performance, focusing on the latter as the primary explanatory variable. Given that patents often yield economic benefits to firms even before they are officially granted, measuring innovation performance in a timely manner relies on the number of corporate patent applications (Wang et al., 2023). Green innovation performance is assessed using the number of green patent applications, while traditional innovation performance is determined by subtracting green patent applications from the total number of patent applications. To reduce data dispersion, the natural logarithm of the number of patent applications is employed (Jiang et al., 2020).

#### 4.2.2 Explanatory variable

##### 4.2.2.1 Environmental management system certification (ISO)

This variable is binary, signifying whether a company holds ISO14001 certification in a given year. A value of 1 is assigned to companies with valid ISO14001 certification, while a value of 0 represents companies without certification or with invalid certification (Dominguez et al., 2016).

#### 4.2.3 Mediating variable

##### 4.2.3.1 Social responsibility disclosure (Csr)

Social responsibility disclosure is assessed using data derived from the social responsibility ratings published by Hexun.com. This evaluation system relies on stakeholder theory to calculate scores based on weighted allocations specific to different industries.

#### 4.2.4 Moderating variables

##### 4.2.4.1 Enterprise size (Size)

Depending on the research objectives and data availability, several metrics are relevant for measuring firm size. Prior studies frequently employ three key indicators: sales, number of employees, and total assets. Other indicators, such as cost of sales, number of subsidiaries, market value of stocks and bonds, and enterprise added value, have also been used to gauge enterprise scale (George et al., 2021; Cheng et al., 2022; Jarrod et al., 2022). In this study, we adopt the logarithm of total assets as our size measurement. Total assets represent the resources under a corporation's management, represented as the sum of liabilities and owner's equity.

##### 4.2.4.2 Nature of property rights (Soe)

We classify enterprises as state-owned (Soe = 1) or non-state-owned (Soe = 0).

##### 4.2.4.3 Stock incentive (Stock)

Equity incentive models in practice can be intricate, including various structures such as equity options, restricted shares, performance stocks, and employee stock ownership plans (Jones et al., 2019; Martin et al., 2019). However, regardless of the specific incentive mechanism, changes in ownership stake ultimately reflect the incentive's impact. Following Davidson (2022) as a reference, this study measures the intensity of management equity incentives using the proportion of total shares held by directors, supervisors, and senior managers relative to total share capital.

##### 4.2.4.4 Market competition intensity (HHI)

The Herfindahl index (HHI) serves as a measure of market competition intensity. Grounded in the structure-operate-performance theory, a smaller HHI indicates lower industry concentration and more significant market competition, while a larger HHI signifies greater industry concentration, potentially leading to monopolistic conditions and reduced market competition.

#### 4.2.5 Control variables

This study draws on previous research literature on environmental management system certification and green innovation performance (Feng et al., 2021, 2022; Wang et al., 2021, 2022; Wan et al., 2022), and the control variables selected according to the content and purpose of this paper include: research and development investment (Rd), measured using the ratio of research and development investment to main business revenue; executive overseas experience (Ovesea), measured using the ratio of 1 when the executive has overseas experience and 0 otherwise; independent (Board), measured by the ratio of the number of independent directors to the number of directors on the board of directors; the balance sheet ratio (Lev), measured by the ratio of total liabilities to total assets; the current ratio (Cur), measured by the ratio of total current assets to total current liabilities; the profitability (Roa), measured by the ratio of net income to total assets. Capital intensity (Fixed), measured by the ratio of fixed assets to total assets; equity concentration (Top), measured by the percentage of shares held by the largest shareholder; growth capacity (Grow), selected the growth rate of operating revenue as a proxy variable; and also controlled for industry and year.

### 4.3 Regression model

Multiple regression models (Models 1, 2, and 3) are constructed to test Hypotheses 1, followed by Models 4 and 5 to test Hypothesis 2. The analysis begins by examining the impact of environmental management system certification on innovation performance and subsequently dissects traditional innovation performance and green innovation performance in separate groups. The study selects green innovation performance, which exhibits a more significant association with environmental management system certification, for deeper investigation. The mediating effect of social responsibility disclosure is then explored. Finally, Models 6, 7, and 8 are formulated to assess Hypotheses 3 to 6, scrutinizing the moderating influences of firm size, property rights nature, equity incentives, and market competition intensity on the mediating pathway.


(1) $$\begin{array}{l} Tpatant=\alpha 0+\alpha 1\;ISO+\sum Controls+Industry+Year+\varepsilon \end{array}$$

(2) $$\begin{array}{l} Bpatant=\pi 0+\pi 1\;ISO+\sum Controls+Industry+Year+\varepsilon \end{array}$$

(3) $$\begin{array}{l} Gpatant=\lambda 0+\lambda 1\;ISO+\sum Controls+Industry+Year+\varepsilon \end{array}$$

(4) $$\begin{array}{l} Csr=\beta 0+\beta 1\;ISO+\sum Controls+Industry+Year+\varepsilon \end{array}$$

(5) $$\begin{array}{l} Gpatant=\gamma 0+\gamma 1\;ISO+\gamma 2Csr+\sum Controls+Industry+Year+\varepsilon \end{array}$$

(6) $$\begin{array}{l} Gpatant=\theta 0+\theta 1\;ISO+\theta 2W+\theta 3\;ISO\times W+\sum Controls+Industry+Year+\varepsilon \end{array}$$

(7) $$\begin{array}{l} Csr=\vartheta \;0+\vartheta \;1\;ISO+\vartheta \;2W+\vartheta \;3\;ISO\times W+\sum Controls+Industry+Year+\varepsilon \end{array}$$

(8) $$\begin{array}{l} Gpatant=\mu 0+\mu 1\;ISO+\mu 2W+\mu 3\;ISO\times W+\mu 4Csr+\mu 5Csr\times W+\sum Controls+Industry+Year+\varepsilon \end{array}$$

W in the model is each moderating variable, Controls represents a set of control variables, and ε is the error term.

## 5 Results

### 5.1 Descriptive statistics and correlation analysis of variables

Table 1 presents the descriptive statistics for the study's primary variables. The mean innovation performance score is 3.341, with a standard deviation of 1.342. The range of innovation performance spans from 0.693 to 7.101, highlighting significant variation among sample companies. Traditional innovation performance has a mean of 3.187, while green innovation performance averages 1.106. This suggests that traditional innovation performance surpasses green innovation performance on average. Environmental management system certification, with a mean value of 0.285, indicates that only 28.5% of the sample companies possess such certification, signifying its relatively low prevalence. Social responsibility disclosure scores exhibit a mean of 24.435, with a median score of 21.750, indicating a generally low level of disclosure.

**Table 1:** Descriptive statistics of the main variables.

**Variables**	***N***	**Mean**	**P50**	**S.D**.	**Min**	**Max**
Tpatant	3.949	3.341	3.258	1.342	0.693	7.101
Bpatant	3.949	3.187	3.135	1.396	0	7.055
Gpatant	3.949	1.106	0.693	1.214	0	4.673
ISO	3.949	0.285	0	0.452	0	1
Csr	3.949	24.435	21.750	14.569	1.390	74.160
Size	3.949	12.792	12.629	1.187	10.862	16.518
Soe	3.949	0.309	0	0.462	0	1
Stock	3.949	0.165	0.037	0.208	0	0.683
HHI	3.949	0.165	0.109	0.164	0.023	1
Rd	3.949	0.049	0.039	0.041	0.001	0.239
Ovesea	3.949	0.253	0	0.435	0	1
Board	3.949	0.373	0.333	0.052	0.333	0.571
Lev	3.949	0.374	0.360	0.191	0.045	0.822
Cur	3.949	2.981	1.903	3.186	0.480	20.451
Roa	3.949	0.049	0.043	0.045	−0.073	0.192
Fixed	3.949	0.216	0.192	0.137	0.009	0.616
Top	3.949	0.339	0.322	0.143	0.083	0.729
Grow	3.949	0.155	0.099	0.302	−0.401	1.705

Table 2 displays the results of the correlation analysis among the main study variables. The highest absolute correlation coefficient observed is 0.508, and the variance inflation factor (VIF) for each variable is <10, indicating no issues of multicollinearity. Furthermore, green innovation performance shows significant correlations with all primary variables, and the explanatory variables also exhibit significant correlations with the mediating variables, supporting the validity of variable selection.

**Table 2:** Pearson correlation coefficients among variables.

	**Gpatant**	**ISO**	**Csr**	**Size**	**Soe**	**Stock**	**HHI**
Gpatant	1	0.038^**^	0.055^***^	0.486^***^	0.201^***^	−0.161^***^	0.077^***^
ISO	0.038^**^	1	0.158^***^	0.045^***^	0.050^***^	−0.052^***^	0.007
Csr	0.055^***^	0.158^***^	1	0.160^***^	0.072^***^	−0.02	0.012
Size	0.486^***^	0.045^***^	0.160^***^	1	0.391^***^	−0.401^***^	0.067^***^
Soe	0.201^***^	0.050^***^	0.072^***^	0.391^***^	1	−0.508^***^	−0.028^*^
Stock	−0.161^***^	−0.052^***^	−0.02	−0.401^***^	−0.508^***^	1	−0.009
HHI	0.077^***^	0.007	0.012	0.067^***^	−0.028^*^	−0.009	1

^***^, ^**^ and ^*^ indicate significant at the 1, 5, and 10% significance levels, respectively.

### 5.2 Analysis of regression

#### 5.2.1 Main effects test

To evaluate Hypothesis H1, regression analyses were carried out, incorporating innovation performance, traditional innovation performance, and green innovation performance as explanatory variables. Environmental management system certification, alongside several control variables, was included as explanatory variables. The regression results are presented in Table 3. Notably, the coefficients of ISO were found to be 0.082 and 0.096 when innovation performance and green innovation performance were considered as explanatory variables, respectively. Both coefficients were statistically significant at the 5% significance level. However, the coefficient was 0.057 and not statistically significant when applied to traditional innovation performance. In essence, this indicates that environmental management system certification has the potential to enhance innovation performance. Moreover, when compared with traditional innovation performance, it was observed that environmental management system certification can more effectively promote green innovation performance. This suggests that after certification, businesses are more inclined to engage in green innovation activities aimed at mitigating environmental risks, pollution, and other adverse resource utilization effects. As a result, their green innovation performance improves, thereby corroborating Hypothesis H1. Subsequent investigations will continue to focus on environmental management system certification and its impact on green innovation performance.

**Table 3:** Main effects test and mediating effects test.

	**Model 1**	**Model 2**	**Model 3**	**Model 4**	**Model 5**
	**Tpatant**	**Bpatant**	**Gpatant**	**Csr**	**Gpatant**
ISO	0.082^**^	0.057	0.096^**^	5.602^***^	0.038
	−0.042	−0.044	−0.038	−0.453	−0.039
Csr					0.010^***^
					−0.001
_cons	1.531^***^	1.394^***^	−0.237	14.725^***^	−0.388^**^
	−0.182	−0.193	−0.167	−1.985	−0.167
Controls	Yes	Yes	Yes	Yes	Yes
Industry	Yes	Yes	Yes	Yes	Yes
Year	Yes	Yes	Yes	Yes	Yes
N	3.949	3.949	3.949	3.949	3.949
R^2^	0.259	0.233	0.236	0.253	0.247

^***^, ^**^ and ^*^ indicate significant at the 1, 5, and 10% significance levels, respectively.

#### 5.2.2 Mediating effect test

To examine the mediating effect of social responsibility disclosure, this study follows a three-step approach. Firstly, the preceding analysis has already established that environmental management system certification facilitates corporate green innovation performance. Secondly, the influence of environmental management system certification on social responsibility disclosure is investigated. In Model 4, the coefficient for environmental management system certification and social responsibility disclosure is determined to be 5.602, and this coefficient is statistically significant at the 1% significance level. This finding substantiates the enhancing impact of environmental management system certification on social responsibility disclosure. Finally, the mediating variable is introduced. The regression results of Model 5 reveal that the coefficient for environmental management system certification becomes 0.038 and is no longer statistically significant, whereas the coefficient for social responsibility disclosure is 0.01 and passes the significance test at the 1% level. Consequently, the mediating effect of social responsibility disclosure is significant, amounting to 58.36%. In essence, this means that 58.36% of the influence of environmental management system certification on green innovation performance is mediated through the effect of social responsibility disclosure. The ultimate outcome suggests that, following certification, enterprises tend to bolster their social responsibility disclosure efforts with the aim of enhancing their reputation. This, in turn, biases stakeholders' decision-making processes in a manner conducive to boosting green innovation performance, thus confirming Hypothesis H2.

#### 5.2.3 Mediating effects test with moderation

The outcomes of the regulatory impact evaluation for enterprise scale are presented in Table 4. Firstly, the coefficient of the main effect interaction term was found to be statistically insignificant. However, in Model 7, the coefficient of the interaction term was positive and demonstrated statistical significance at the 5% level. This suggests that the regulatory effect of enterprise scale operates in the first part of the intermediary path, effectively establishing the intermediary effect with regulation. In essence, this means that larger-scale enterprises tend to prioritize their image construction and actively disclose social responsibility information after obtaining environmental management system certification. This proactive stance benefits them in acquiring innovation resources, thereby enhancing their green innovation performance. Additionally, the economies of scale effect, stemming from scale expansion that reduces various costs, including disclosure costs, positively contributes to the improvement of green innovation performance. Consequently, larger enterprises experience a more pronounced promotional effect of environmental management system certification on social responsibility disclosure, which, in turn, enhances their green innovation performance, thus confirming Hypothesis H3.

**Table 4:** Mediating effect test with moderation.

	**Firm size**			**Nature of ownership**			**Equity incentives**			**Market competition**		
	**Gpatant**	**Csr**	**Gpatant**	**Gpatant**	**Csr**	**Gpatant**	**Gpatant**	**Csr**	**Gpatant**	**Gpatant**	**Csr**	**Gpatant**
ISO	−0.047	−4.79	0.011	0.160^***^	5.427^***^	0.11^**^	0.011	4.757^***^	−0.035	0.129^**^	4.439^***^	0.107
	−0.388	−4.868	−0.391	−0.046	−0.548	−0.047	−0.048	−0.567	−0.048	−0.054	−0.642	−0.055
Csr			−0.008			0.009^***^			0.01^***^			0.008^***^
			−0.011			−0.002			−0.002			−0.002
Size	0.469^***^	3.161^***^	0.443^***^									
	−0.019	−0.24	−0.029									
ISO × size	0.009	0.795^**^	0.004									
	−0.03	−0.377	−0.03									
Csr × size			0.001									
			−0.001									
Soe				0.449^***^	3.654^**^	0.435^***^						
				−0.048	−0.567	−0.072						
ISO × Soe				−0.242^***^	0.082	−0.238^***^						
				−0.08	−0.95	−0.08						
Csr × Soe						−0.001						
						−0.002						
Stock							−0.733^***^	−6.539^**^	−0.635^**^			
							−0.103	−1.222	−0.178			
ISO × Stock							0.509^***^	5.1^**^	0.47^**^			
							−0.186	−2.211	−0.189			
Csr × Stock									−0.002			
									−0.006			
HHI										0.233	−1.195	−0.066
										−0.152	−1.808	−0.219
ISO × HHI										−0.209	7.002^**^	−0.432
										−0.232	−2.754	−0.243
Csr × HHI												0.014
												−0.007
_cons	−5.069^***^	−24.51^***^	−4.769^***^	−0.300^**^	7.944^***^	−0.256	−0.103	9.125^***^	−0.114		8.193^***^	−0.205
	−0.261	−3.287	−0.377	−0.166	−1.938	−0.163	−0.167	−1.953	−0.165	−0.169	−1.97	−0.167
Controls	Yes	Yes	Yes	Yes	Yes	Yes	Yes	Yes	Yes	Yes	Yes	Yes
Industry	Yes	Yes	Yes	Yes	Yes	Yes	Yes	Yes	Yes	Yes	Yes	Yes
Year	Yes	Yes	Yes	Yes	Yes	Yes	Yes	Yes	Yes	Yes	Yes	Yes
*N*	3.949	3.949	3.949	3.949	3.949	3.949	3.949	3.949	3.949	3.949	3.949	3.949
*R* ^2^	0.357	0.3	0.358	0.254	0.264	0.262	0.246	0.258	0.256	0.236	0.254	0.256

^***^, ^**^ and ^*^ indicate significant at the 1, 5, and 10% significance levels, respectively.

The moderating influence of the nature of property rights on the main effect and the mediating path was analyzed in accordance with Hypothesis H4. According to the analysis of the test results in Model 6, the coefficient of the interaction term was −0.242, signifying significance at the 1% level. This indicates that the facilitation effect is more pronounced in non-state enterprises. While the coefficients of the interaction term in the intermediary path were not statistically significant, it was observed that the intermediary effect of the nature of property rights with regulation is not valid. This suggests that enterprises, regardless of their property rights nature, tend to engage in environmental management work once they have obtained environmental management system certification. Property rights nature does not substantially impact the mediation path. However, the coefficient of the direct effect interaction term was −0.238 and demonstrated statistical significance at the 1% level. This implies that the moderating influence of property rights nature primarily operates on the direct path of environmental management system certification and green innovation performance. In state-owned enterprises, where resource acquisition costs are relatively low, the marginal utility of voluntary environmental management in optimizing resource allocation to enhance green innovation performance is weak. Conversely, non-state-owned enterprises rely on environmental management system certification to boost their green innovation performance. As a result, the moderating effect of environmental management system certification on the social responsibility disclosure path is not significant in non-state-owned enterprises, and the direct enhancement of green innovation performance is more pronounced. These findings affirm Hypothesis H4.

The moderating influence of equity incentives on the mediating path was investigated in accordance with Hypothesis H5. As per the analysis of the test results in Model 6, the coefficient of the interaction term was 0.509 and demonstrated statistical significance at the 1% level. This suggests that stronger equity incentives within an enterprise can amplify the role of environmental management system certification in enhancing green innovation performance. In Model 7, the coefficient of the interaction term was significantly positive, thus establishing a moderating mediating effect. Consequently, it can be inferred that corporate equity incentives heighten management's awareness of social responsibility, align corporate sustainable development with their individual interests, and alleviate the principal-agent problem. A higher degree of equity incentives fosters management decisions favoring social responsibility disclosure when the enterprise is certified. This, in turn, enhances the enterprise's green innovation performance, thus confirming Hypothesis H5.

The moderating influence of market competition intensity on the intermediation path was assessed in line with Hypothesis H6. The results indicated that, in Model 6, the coefficient of the interaction term was not statistically significant. However, in Model 7, the coefficient of the interaction term was 7.002 and demonstrated statistical significance at the 5% level. This suggests that the moderating effect of market competition intensity is indeed present. When the Herfindahl index (HHI) is larger, indicating weaker market competition intensity, the enhancement effect of environmental management system certification on social responsibility disclosure is more pronounced. This, in turn, promotes green innovation performance, thereby verifying Hypothesis H6. In scenarios of high market competition intensity, the short-term profit-seeking behavior of enterprises tends to influence corporate social responsibility disclosure decisions. Even after obtaining environmental management system certification, they factor in the cost of social responsibility disclosure, which inhibits the promotion effect of environmental management system certification on social responsibility disclosure. In contrast, when competition intensity is lower, enterprises prioritize environmental management, promote social responsibility disclosure, and consequently, bolster green innovation performance.

The comparison results of specific hypotheses and conclusions are shown in Table 5.

**Table 5:** Comparison of hypotheses and conclusions.

**Hypothese**	**Conclusion**
H1a: Environmental management system certification positively influences enterprise innovation performance.	Environmental management system certification exerts an augmenting influence on innovation performance, with its effect on green innovation performance being notably more pronounced when contrasted with conventional innovation performance.
H1b: Environmental management system certification has a more pronounced positive effect on enterprise green innovation performance compared to traditional innovation performance.		
H2: Environmental management system certification enhances corporate green innovation performance by promoting social responsibility disclosure.		Hypothesis is valid. Environmental management system certification exercises its influence on green innovation performance by virtue of social responsibility disclosure.
H3: The larger the firm size, the more it can enhance the impact of environmental management system certification on social responsibility disclosure, thus amplifying its influence on green innovation performance.		Hypothesis is valid. The magnitude of a firm's size, the extent of equity incentives extended, and the intensity of market competition faced collectively amplify the promotional effect of environmental management system certification on social responsibility disclosure, consequently reinforcing its enhancement effect on green innovation performance.
H4: In comparison to state-owned enterprises, non-state-owned enterprises are more inclined to witness the promotion of social responsibility disclosure through environmental management system certification, resulting in a more pronounced effect on green innovation performance.		
H5: The stronger the equity incentive the more it promotes the enhancing effect of environmental management system certification on social responsibility disclosure, and thus on green innovation performance.		
H6: Diminished market competition intensity positively correlates with an augmented influence of environmental management system certification on social responsibility disclosure, consequently amplifying its impact on green innovation performance.		

### 5.3 Robustness testing

#### 5.3.1 Endogeneity

The previous results suggest that environmental management system certification holds the potential to enhance firms' green innovation performance. However, it's essential to consider the possibility that firms with lower green innovation performance might be using environmental management system certification as a means to finance their green innovation activities, thus introducing endogeneity concerns. To address this issue, we conducted a robust heteroskedasticity test using the Durbin-Wu-Hausman (DWH) method, yielding a *p*-value below 0.05, which rejects the initial hypothesis of exogeneity and indicates the presence of endogeneity problems. In response, we employed instrumental variables through a two-stage least squares (2SLS) approach. In the first stage, we utilized instrumental variables—specifically, “Disclose” and “Action with ISO.” The results, as shown in Table 6, demonstrated significant coefficients of 0.246 and 0.091, respectively, both at the 1% significance level. Subsequently, in the second stage, the coefficient for “ISO with Gpatant” was 1.989, also passing the 1% significance threshold. Furthermore, we conducted weak instrumental variables tests and over-identification tests to validate the instrumental variables. The weak instrumental variables test yielded an *F*-value exceeding 10, while the over-identification test resulted in a *p*-value of 0.880, surpassing 0.05. These outcomes indicate the absence of weak instrumental variables and affirm the original hypothesis that all variables are exogenous. Overall, these results confirm that environmental management system certification maintains its capacity to promote green innovation performance even after accounting for endogeneity issues, consistent with our prior findings.

**Table 6:** Endogeneity test and robustness test.

	**Two-stage regression of instrumental variables**		**Substitution of dependent variables and lagged regression**			**Further analysis and testing**			
	**First Stage Regression**	**Second Stage Regression**	**Substitution of dependent variable**	**Lag one period**	**Lag two periods**	**Exploratory innovation**	**Utilization innovation**	**High level**	**Low level**
	**ISO**	**Gpatant**	**Gpatant**	**L.Gpatant**	**L2.Gpatant**	**Gpatant**	**Gpatant**	**Gpatant**	**Gpatant**
ISO		1.989^***^	0.140^***^	0.140^***^	0.150^***^	0.091^***^	0.029	0.072	0.122^**^
		−0.182	−0.027	−0.038	−0.043	(−0.033)	(−0.031)	(−0.058)	(−0.053)
Disclose	0.246^***^								
	−0.019								
Action	0.091^***^								
	−0.023								
_cons	0.381^***^	−0.824^***^	0.065	0.078	0.118	−0.422^***^	−0.243^*^	0.033	−0.06
	−0.067	−0.219	−0.119	−0.121	−0.191	(−0.142)	(−0.137)	(−0.239)	(−0.224)
Controls	Yes	Yes	Yes	Yes	Yes	Yes	Yes	Yes	Yes
Industry	Yes	Yes	Yes	Yes	Yes	Yes	Yes	Yes	Yes
Year	Yes	Yes	Yes	Yes	Yes	Yes	Yes	Yes	Yes

^***, **^ and ^*^ indicate significant at the 1, 5, and 10% significance levels, respectively.

#### 5.3.2 Substitution of dependent variables

Given that green patents applied for by enterprises do not always lead to eventual authorization, and recognizing the considerable time lag in the patent process, this study sought to investigate the long-term sustainability of the impact of environmental management system (EMS) certification. To achieve this, we substituted the number of green patents authorized by enterprises, introducing one and two-period lags, in place of the number of patent applications. The results of this substitution are presented in Table 6. Remarkably, the regression outcomes align consistently with our earlier findings, thereby providing additional substantiation for Hypothesis H1.

#### 5.3.3 Transformation test method

In pursuit of a comprehensive examination of the mediating effect, our study employed a Bootstrap test. The results, as depicted in Table 7, unveil a 95% confidence interval for the indirect effect of ISO on Gpatant spanning from (0.0408, 0.0785). Importantly, this interval does not encompass 0, decisively confirming the existence of a mediating effect. Additionally, the 95% confidence interval for the direct effect of ISO on Gpatant ranges from (−0.0374, 0.1141), with this interval including 0. This implies that corporate social responsibility (CSR) entirely mediates the effect, accounting for 60.1%. These outcomes robustly corroborate our previous conclusions, providing further support for Hypothesis H2.

**Table 7:** Bootstrap mediating effect test.

**Total effect of ISO on Gpatant**					
**Effect**	**SE**	**t**	**p**	**LLCI**	**ULCI**
0.096	0.0382	2.5152	0.0119	0.0212	0.1709
**Direct effect of ISO on Gpatant**					
**Effect**	**SE**	**t**	**p**	**LLCI**	**ULCI**
0.0383	0.0386	0.9919	0.3213	−0.0374	0.1141
**Indirect effect of ISO on Gpatant**					
**Effect**	**BootSE**		**BootLLCI**		**BootULCI**
0.0577	0.0094		0.0408		0.0785

### 5.4 Further analysis

Our preceding findings conclusively indicate that environmental management system (EMS) certification exerts a significantly positive impact on green innovation performance. In light of these results, we proceeded to delve into the nuances of green innovation performance by categorizing it into two distinct types: exploratory green innovation performance and utilization green innovation performance. Exploratory green innovation represents a strategic avenue for allocating enterprise resources, constituting a pivotal means to bolster a firm's core competitiveness. Conversely, utilization green innovation entails iterative improvements on existing resources with a focus on expanding possibilities on a smaller scale. In the context of enhancing enterprise green innovation performance via EMS certification, our hypothesis posited a predilection toward exploratory green innovation performance. To gauge and validate these distinctions, we quantified exploratory vs. exploitative green innovation performance by employing the natural logarithm of the number of green invention patent applications and green utility model patent applications. The regression results are meticulously documented in Table 6, where the initial two columns offer enlightening insights. Specifically, these results underscore that EMS certification is markedly more inclined to stimulate exploratory green innovation performance as opposed to exploitative green innovation performance.

Our investigation unearthed a profound linkage between the impact of EMS certification on green innovation performance and the prevailing degree of regional economic development. In regions characterized by elevated economic development, enterprises benefit from reduced innovation financing costs, facilitating their access to resources conducive to green innovation performance, even in the absence of EMS certification. Conversely, regions grappling with lower levels of economic development present enterprises with financing challenges. Under these circumstances, leveraging EMS certification to fulfill social responsibility emerges as a strategy to bolster stakeholders' confidence, mitigate innovation financing risks, and streamline the acquisition of green innovation-related resources, all while minimizing capital costs. Our metric for gauging regional economic development rested upon regional GDP per capita. Specifically, regions boasting a per capita GDP surpassing the sample median were classified as experiencing high economic development, while those falling below the median were deemed to exhibit low economic development. The regression outcomes, systematically presented in Table 6, offer compelling insights. In regions characterized by high economic development, the ISO regression coefficient proves statistically insignificant. Conversely, regions grappling with low economic development levels yield a significantly positive ISO regression coefficient. These outcomes decisively affirm that EMS certification is substantially more effective in propelling green innovation performance within regions marked by lower economic development levels.

## 6 Conclusions and discussions

Environmental management system certification, functioning as a voluntary environmental regulatory instrument, exerts a positive influence on the green innovation performance of enterprises. While previous studies have delved into the impact of environmental management system certification on the advancement of corporate green innovation performance from the vantage point of innovation theory, they have left notable gaps in scrutinizing the pathways through which these impacts manifest. Consequently, this study delves into the nuanced, indirect effect of environmental management system certification on corporate green innovation performance, within the contextual framework of stakeholder theory, signaling theory, and reputation theory, among others. The empirical analysis yields the following salient conclusions: (i) Environmental management system certification exerts an augmenting influence on innovation performance, with its effect on green innovation performance being notably more pronounced when contrasted with conventional innovation performance. (ii) Environmental management system certification exercises its influence on green innovation performance by virtue of social responsibility disclosure. (iii) The magnitude of a firm's size, the extent of equity incentives extended, and the intensity of market competition faced collectively amplify the promotional effect of environmental management system certification on social responsibility disclosure, consequently reinforcing its enhancement effect on green innovation performance. (iv) The moderating impact of property rights nature on the mediating path does not achieve statistical significance. However, the moderating effect on the direct influence path is indeed substantial; this implies that the direct promotional effect of environmental management system certification on green innovation performance is markedly more conspicuous in non-state enterprises. (v) Environmental management system certification exerts a more pronounced influence on enhancing green innovation performance within regions characterized by a lower level of economic development. Furthermore, in comparison to exploitative green innovation performance, it significantly fosters exploratory green innovation performance. These findings collectively contribute to our understanding of the multifaceted relationship between environmental management system certification and green innovation performance.

### 6.1 Policy applications

This study extends the purview of environmental protection to encompass the realm of corporate green innovation through social responsibility disclosure. This expansion augments the existing body of research and furnishes valuable practical insights: (i) Promoting Active Pursuit of Environmental Management System Certification: Encouraging enterprises to proactively seek environmental management system certification is essential. This approach harnesses their subjective initiative to assume societal responsibility. Consequently, it not only contributes to the preservation of regional ecological environments but also enhances corporate green innovation performance. This 2-fold benefit ensures the normalization and long-term effectiveness of environmental protection endeavors. (ii) Energizing State-Owned Enterprises: The findings underscore the significance of invigorating state-owned enterprises. By leveraging environmental management to fuel enthusiasm for green innovation, these enterprises can achieve sustainable development. (iii) Strengthening Equity Incentive Mechanisms: To motivate management to embrace corporate and social development as their responsibility, enhancing equity incentive mechanisms is paramount. Aligning management's interests with environmental management systems can stimulate green innovation and economic development. (iv) Mitigating Backlash from Excessive Competition: To forestall adverse repercussions stemming from excessive competition, optimizing the allocation of market resources is pivotal. This optimization should encompass the utilization of environmental management strategies to promote green innovation development, thereby fostering healthy market competition.

### 6.2 Limitations and future directions

While this study holds significant implications, it is not without limitations. Firstly, from a research perspective, this paper primarily explores the influence mechanism of environmental management on green innovation through the lens of environmental management system certification—a voluntary, participatory environmental regulation. The study has yet to delve into other forms of environmental regulation and governance mechanisms. Future research should broaden its scope to encompass these aspects. Secondly, this study conducts an analysis of environmental information disclosure within developing countries. It's important to recognize that our findings may not be universally applicable to countries with different cultural climates and economic systems. These findings should be subjected to further scrutiny to ascertain their reproducibility and generalizability across different research frameworks. Addressing these research gaps, future studies may prioritize the following areas: (i) Exploring Environmental Systems and Governance Mechanisms: Future research can further investigate how environmental systems, corporate governance, and internal controls can be harnessed to maximize the utility of environmental management system certification in enhancing innovation output. Such studies can provide additional theoretical underpinnings and policy insights for the implementation of innovation-driven strategies. (ii) Investigating Imitation and Spillover Effects: In regions where data availability permits, it is valuable to continue exploring the impact of environmental management system certification on the green innovation performance of enterprises in developed countries. Such research can shed light on potential imitation and spillover effects, contributing to a more comprehensive understanding of the subject.

## Data availability statement

The raw data supporting the conclusions of this article will be made available by the authors, without undue reservation.

## Author contributions

JZ: Supervision, Writing – review & editing. MW: Formal analysis, Writing – original draft, Writing – review & editing. ML: Writing – review & editing.
